# Single-cell RNA sequencing reveals hypoxia-associated stress response signatures in the immune and stromal microenvironment of laryngeal squamous cell carcinoma

**DOI:** 10.3389/fimmu.2026.1850654

**Published:** 2026-08-04

**Authors:** Huihui Du, Yating Wang, Renyu Lin, Jiaqi Yu, Kuaiquan Zhu

**Affiliations:** 1 Otorhinolaryngology, Wenzhou Hospital of Integrated Traditional Chinese and Western Medicine, Wenzhou, Zhejiang, China; 2 Department of Otolaryngology, The First Affiliated Hospital of Wenzhou Medical University, Wenzhou, Zhejiang, China

**Keywords:** biomarkers, HIF-1 signaling, hypoxia, immune enrichment, laryngeal squamous cell carcinoma, myeloid, single-cell RNA sequencing, stress response

## Abstract

**Background:**

Laryngeal squamous cell carcinoma (LSCC) remains a major clinical challenge with limited therapeutic options and unsatisfactory outcomes. Hypoxia-induced stress responses are known to contribute to tumor progression, immune remodeling, and therapeutic resistance. However, the cell type-specific hypoxia-associated transcriptional programs within the immune and stromal microenvironment of LSCC remain incompletely understood.

**Methods:**

We analyzed an immune-enriched LSCC single-cell RNA sequencing dataset (GSE150321, GSM4546858), generated by CD45-based magnetic bead sorting, to characterize hypoxia-associated stress response signatures across immune and stromal cell populations. The workflow included quality control, dimensionality reduction, unsupervised clustering, cell type annotation, pathway enrichment, hypoxia score assessment, robust rank aggregation, pseudotime trajectory inference, and cell–cell communication analysis. Representative hypoxia-associated genes were further validated experimentally in LSCC-related cell models.

**Results:**

Nine major cell populations were identified, including T cells, epithelial cells, B cells, macrophages, dendritic cells, cycling cells, fibroblasts, mast cells, and endothelial cells. Hypoxia-associated pathway activity was broadly enriched, with particularly strong activation in myeloid populations. Slingshot analysis using macrophages as the trajectory root revealed a monocyte-to-macrophage maturation trajectory, along which hypoxia scores were highest in macrophages and decreased toward lymphoid cells (Spearman R = −0.271, P = 1.78 × 10^-^¹^7^). Myeloid-specific hypoxia-associated genes, including PLA2G7, CD163, and APOC1, were identified. Robust rank aggregation further highlighted candidate hypoxia-associated genes such as HIF1A, CXCL8, S100A8, and ENO1. Experimental validation in THP-1 cells confirmed hypoxia-induced upregulation of these genes, and ELISA showed increased CXCL8 secretion under hypoxia (985 ± 87 vs. 215 ± 34 pg/mL, P < 0.001). In contrast, the novel myeloid-specific genes showed negligible expression in Hep-2 epithelial cells, supporting their cell-type specificity.

**Conclusions:**

This study characterizes hypoxia-associated transcriptional programs within the immune and stromal microenvironment of LSCC rather than providing a comprehensive malignant epithelial tumor atlas. The findings highlight myeloid-centered hypoxia adaptation as potential sources of biomarker candidates and therapeutic insight for LSCC. THP-1 monocytic cell validation provides functional support for myeloid-centered hypoxia adaptation.

## Introduction

Laryngeal squamous cell carcinoma (LSCC) represents one of the most prevalent head and neck malignancies, accounting for approximately 95% of all laryngeal cancers and significantly contributing to cancer-related morbidity and mortality worldwide (1). Despite advances in surgical techniques, radiation therapy, and chemotherapy protocols, the five-year survival rate for LSCC patients has remained relatively stagnant over the past several decades, particularly for advanced-stage disease (2). The heterogeneous nature of LSCC, combined with its propensity for local recurrence and distant metastasis, underscores the urgent need for novel therapeutic targets and more precise diagnostic and prognostic biomarkers (3).Hypoxia, characterized by insufficient oxygen supply to meet cellular metabolic demands, represents a fundamental feature of the tumor microenvironment that profoundly influences cancer progression, therapeutic resistance, and patient outcomes (4). In LSCC, hypoxic conditions arise due to rapid tumor growth, abnormal vascularization, and increased oxygen consumption, creating a challenging microenvironment that promotes aggressive tumor behavior (5). The cellular response to hypoxia is primarily mediated through hypoxia-inducible factors (HIFs), particularly HIF-1α, which act as master transcriptional regulators orchestrating adaptive responses including angiogenesis, metabolic reprogramming, epithelial-mesenchymal transition, and immune evasion (6, 7).The tumor microenvironment in LSCC comprises a complex ecosystem of diverse cell types, including malignant epithelial cells, immune cell populations, stromal fibroblasts, endothelial cells, and various progenitor cell populations (8, 9). Each of these cellular components responds differently to hypoxic stress, contributing to the overall tumor phenotype through distinct molecular mechanisms and intercellular communication networks (10). Traditional bulk RNA sequencing approaches, while valuable, lack the resolution to dissect cell type-specific responses to hypoxic stress, potentially masking critical biological insights that could inform therapeutic strategies.Single-cell RNA sequencing (scRNA-seq) has emerged as a powerful technology that enables comprehensive characterization of cellular heterogeneity and transcriptional dynamics at unprecedented resolution (11). This approach provides unique opportunities to investigate how different cell types within the tumor microenvironment respond to hypoxic stress, identify cell type-specific biomarkers, and uncover novel therapeutic targets (12). Recent applications of scRNA-seq in cancer research have revealed previously unappreciated cellular diversity and have provided new insights into tumor progression, metastasis, and therapeutic resistance mechanisms (13, 14).The identification of hypoxia-associated biomarkers in LSCC has significant clinical implications for patient stratification, treatment selection, and monitoring therapeutic responses. Current clinical biomarkers for LSCC are limited and often fail to capture the complex molecular heterogeneity underlying disease progression and treatment resistance (15). Cell type-specific hypoxia-responsive gene signatures could provide more precise tools for predicting patient outcomes and identifying individuals who might benefit from hypoxia-targeted therapies or modified treatment regimens. In this study, we employed comprehensive single-cell RNA sequencing analysis to investigate hypoxia-associated stress response signatures across distinct cellular populations in laryngeal squamous cell carcinoma. Our integrated analytical approach, combining rigorous quality control, advanced dimensionality reduction techniques, cell type identification, pathway enrichment analysis, and robust statistical validation, aims to provide a detailed molecular atlas of hypoxic stress responses in LSCC and identify novel biomarker candidates with potential clinical utility. Through this work, we seek to advance our understanding of the complex interplay between hypoxia and cellular heterogeneity in LSCC and provide a foundation for developing more effective, personalized therapeutic strategies for this challenging malignancy. In addition, *in vitro* validation was performed to assess the expression and functional relevance of selected hypoxia-associated genes in LSCC cells.

## Methods

### KEGG pathway enrichment analysis

Comprehensive KEGG pathway enrichment analysis was performed on differentially expressed genes to identify key biological processes implicated in hypoxia-associated stress responses in laryngeal squamous cell carcinoma. The analysis focused on identifying the most significantly enriched pathways, with particular attention to the Ras signaling pathway, endocytosis, and lipid metabolism pathways including alpha-linolenic acid and linoleic acid metabolism. The HIF-1 signaling pathway was specifically examined to confirm the involvement of hypoxia-inducible factor-mediated transcriptional responses in disease pathogenesis. Visualization methods included circular plots to demonstrate the comprehensive distribution of enriched pathways across major functional categories, bubble plot analysis to illustrate the relationship between pathway significance and gene enrichment ratios, and systematic classification charts showing KEGG annotations across major biological process categories. For KEGG pathway enrichment, pathways with adjusted P<0.05 were statistically enriched.

### Single-cell RNA sequencing and quality control

The raw and processed single-cell RNA sequencing data used in this study are publicly available from the Gene Expression Omnibus (GEO) database under accession number GSE150321. Single-cell RNA sequencing data underwent rigorous quality control analysis to ensure robust cellular profiles suitable for hypoxia-related stress response biomarker discovery. Quality control metrics included assessment of detected gene features per cell (nFeature_RNA), with most cells containing 2000–4000 genes, and RNA count distribution (nCount_RNA) evaluation showing appropriate sequencing depth ranging from 5000–25000 UMIs per cell. Mitochondrial gene percentage analysis (percent.mt) was conducted to identify viable cells and avoid contamination from dying or stressed cells that could confound hypoxia-related gene expression patterns. Correlation analysis between the number of detected genes and total RNA counts was performed to validate technical sequencing quality. Outlier cells with extremely high or low gene counts were flagged and removed to maintain data integrity. Violin plots and density plots were used to visualize the distribution characteristics of quality metrics across the cellular population.

### Principal component analysis and dimensionality reduction

Principal component analysis was implemented to identify distinct transcriptional signatures and the most informative genes for hypoxia-associated biomarker discovery. Gene loading plots for the first principal component (PC_1) were generated to identify genes contributing most significantly to the primary axis of variation, including CXCL1, CXCL8, and other inflammatory and stress response markers. Multi-dimensional PC loading analysis was performed to identify additional gene sets including HIF pathway components and metabolic regulators driving cellular heterogeneity across multiple principal components. PCA scatter plots were used to demonstrate clustering patterns between tumor and normal tissue samples. Heatmap visualization displayed expression patterns of highly variable genes across different cell populations to reveal coordinated expression modules. Variance contribution analysis was conducted using elbow plots and scree plots to determine optimal dimensionality, with the first 15–20 principal components capturing the majority of transcriptional variance in the dataset.

### Single-cell clustering and cell type identification

Unsupervised single-cell clustering analysis was performed to identify distinct cellular populations exhibiting differential responses to hypoxic stress conditions. UMAP (Uniform Manifold Approximation and Projection) dimensionality reduction was applied to visualize well-separated clusters representing diverse cell types, including immune cell populations (CD15+ myeloid cells, myelomonocytic cells, plasmocytes, and NK cells), stromal components (fibroblasts), and progenitor cells (HSPCblast and early myeloid cells). Cell type annotation was conducted through comprehensive marker gene expression analysis, with specific genes showing preferential expression patterns in certain populations. Dot plot visualization was used to display marker gene expression across different cell types, where dot size represented the percentage of cells expressing each gene and color intensity indicated average expression levels. Differentially expressed genes (DEGs) were screened using adjusted P<0.05 (Benjamini–Hochberg FDR correction).

### Cell type-specific pathway enrichment analysis

Comprehensive pathway enrichment analysis was conducted across different cell types to identify cell type-specific activation patterns of stress response and hypoxia-related signaling pathways. HALLMARK pathway analysis was performed to examine immune and metabolic pathway enrichment across major cell types, with particular focus on inflammatory and interferon response pathways including TNF-α signaling via NF-κB and interferon-gamma responses. Hypoxia pathway enrichment was specifically analyzed across multiple cell types, with examination of related stress response pathways including glycolysis, apoptosis, and epithelial-mesenchymal transition (EMT). Heatmap visualization was used to display enrichment scores, with hierarchical clustering applied to group pathways with similar activation patterns across cell types. Statistical significance was assessed using standard enrichment analysis methods with multiple testing correction.

### Robust rank aggregation analysis

Robust rank aggregation (RRA) analysis was implemented to identify consistent hypoxia-associated biomarker candidates across multiple cell types and analytical methods. The systematic ranking approach integrated multiple computational frameworks including DESeq2 and edgeR for differential expression analysis to reduce false discovery rates and enhance the reliability of hypoxia-related gene signatures. Multi-method validation was performed to ensure identified biomarkers represented genuine biological signals rather than method-specific artifacts. Comparative analysis was conducted to examine the distribution and significance of identified biomarkers across different computational methods and cell type classifications. Statistical confidence intervals were calculated to assess the reliability of each cell type’s contribution to the overall biomarker signature, with proportional analysis performed to determine the relative contribution of different cell types to the identified hypoxia-associated gene signatures. For RRA-based candidate gene selection, genes with a combined RRA P-value < 0.05 were considered statistically significant. To define high-confidence hypoxia-associated biomarker candidates, we further prioritized genes ranked within the top 10 by combined RRA P-value and showing consistent upregulation or downregulation across the integrated differential expression analyses. The final candidate genes reported in **Supplementary Table 1** therefore met both statistical significance and rank-based prioritization criteria.

#### Pseudotime trajectory analysis

Pseudotime trajectory inference was performed using Slingshot on immune and myeloid-associated cell populations (macrophages, dendritic cells, cycling immune cells, T/NK cells, B cells, and mast cells). UMAP coordinates from the annotated Seurat object were used as input. Macrophages were designated as the trajectory root, reflecting the monocyte-to-macrophage differentiation hierarchy in which tissue macrophages arise from circulating monocytes. Mast cells represent a distinct terminally differentiated lineage and were assigned accordingly. Hypoxia activity along pseudotime was quantified using the HALLMARK_HYPOXIA gene set, and Spearman correlation assessed the relationship between pseudotime and hypoxia score.

### Experimental validation of selected genes

#### THP-1 cell culture, hypoxia treatment, and ELISA

Human monocytic THP-1 cells (ATCC TIB-202) were cultured in RPMI-1640 supplemented with 10% fetal bovine serum at 37 °C with 5% CO_2_. Hypoxia treatment was performed in a hypoxia workstation (1% O_2_, 5% CO_2_, 94% N_2_) for 24 h. Expression of HIF1A, CXCL8, S100A8, ENO1, PLA2G7, CD163, and APOC1 was measured by qRT-PCR with GAPDH as reference. CXCL8/IL-8 protein in culture supernatants was quantified by ELISA (Human IL-8/CXCL8 Kit, R&D Systems, #D8000C). All assays were performed in triplicate, with statistical comparisons by Student’s t-test.

Human laryngeal squamous carcinoma cells (Hep-2, ATCC CCL-23) and normal laryngeal epithelial cells (Het-1A, ATCC CRL-2692) were cultured in 1% O_2_ hypoxic workstation for 24 h and used for follow-up assays. The expression of HIF1A, CXCL8, S100A8, and ENO1 was measured by quantitative real-time PCR, and Western blot with GAPDH serving as the reference gene. Protein distribution was examined using immunofluorescence staining, followed by nuclear counterstaining with DAPI to visualize cell morphology and localization patterns. To evaluate gene function, Hep-2 cells were transfected with specific small interfering RNAs, and silencing efficiency was assessed at the transcript level. Cell migration was examined using a wound-healing approach, with images captured at baseline and after incubation in serum-free medium. All assays were performed in biological replicates, and statistical comparisons were based on conventional significance thresholds.

## Results

### KEGG pathway enrichment analysis reveals hypoxia-associated stress response signatures in laryngeal squamous cell carcinoma

**Figure 1:**
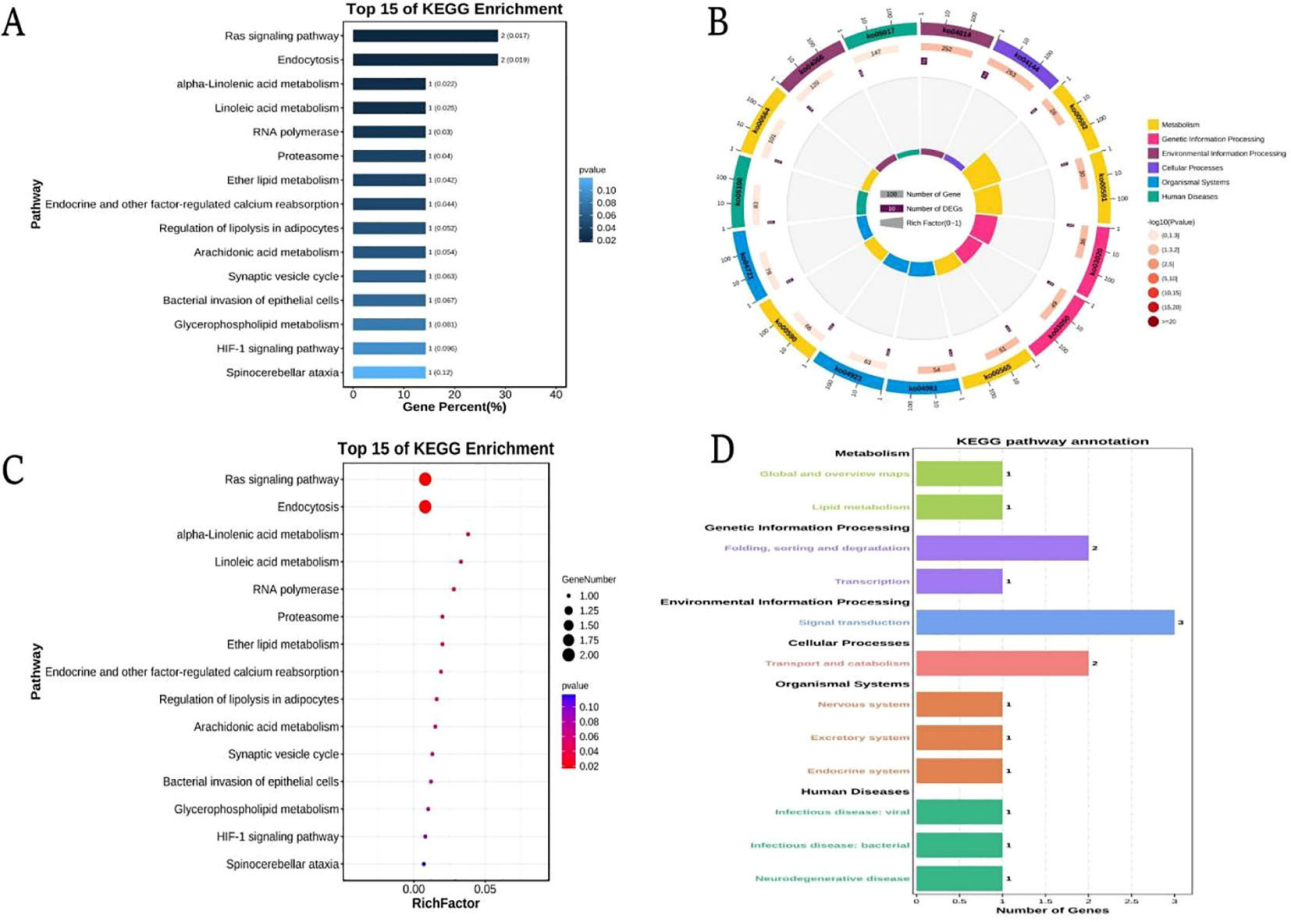
KEGG pathway enrichment analysis reveals hypoxia-associated stress response signatures in laryngeal squamous cell carcinoma. **(A)** Bar plot showing the top 15 most significantly enriched KEGG pathways with gene percentages and p-values. **(B)** Circular plot displaying the overall distribution of KEGG pathway enrichment across different functional categories, with color coding representing major biological processes. **(C)** Bubble plot illustrating the relationship between RichFactor and pathway significance, where bubble size indicates gene number and color represents p-value. **(D)** Horizontal bar chart showing the systematic classification and gene count distribution of KEGG pathway annotations across major biological process categories.

The comprehensive KEGG pathway enrichment analysis of differentially expressed genes identified several key biological processes implicated in hypoxia-associated stress responses in laryngeal squamous cell carcinoma (**Figures 1A–D**). The most significantly enriched pathways included the Ras signaling pathway and endocytosis, followed by lipid metabolism pathways such as alpha-linolenic acid and linoleic acid metabolism, suggesting altered metabolic reprogramming under hypoxic conditions. Notably, the HIF-1 signaling pathway emerged as a significantly enriched pathway, directly confirming the involvement of hypoxia-inducible factor-mediated transcriptional responses in disease pathogenesis.

The circular plot visualization (**Figure 1B**) demonstrates the comprehensive distribution of enriched pathways across major functional categories, with metabolism, genetic information processing, and environmental information processing representing the predominant biological processes. The bubble plot analysis (**Figure 1C**) further illustrates the relationship between pathway significance and gene enrichment ratios, revealing that while some pathways contain fewer genes, they exhibit higher enrichment factors, indicating their critical roles in hypoxic stress adaptation. The systematic classification of KEGG annotations (**Figure 1D**) shows that metabolic processes constitute the largest gene cluster, followed by genetic and environmental information processing pathways, reflecting the cellular reprogramming mechanisms activated during hypoxic stress responses in laryngeal carcinogenesis.

### Single-cell RNA sequencing quality control analysis ensures robust data for hypoxia-associated biomarker discovery

**Figure 2:**
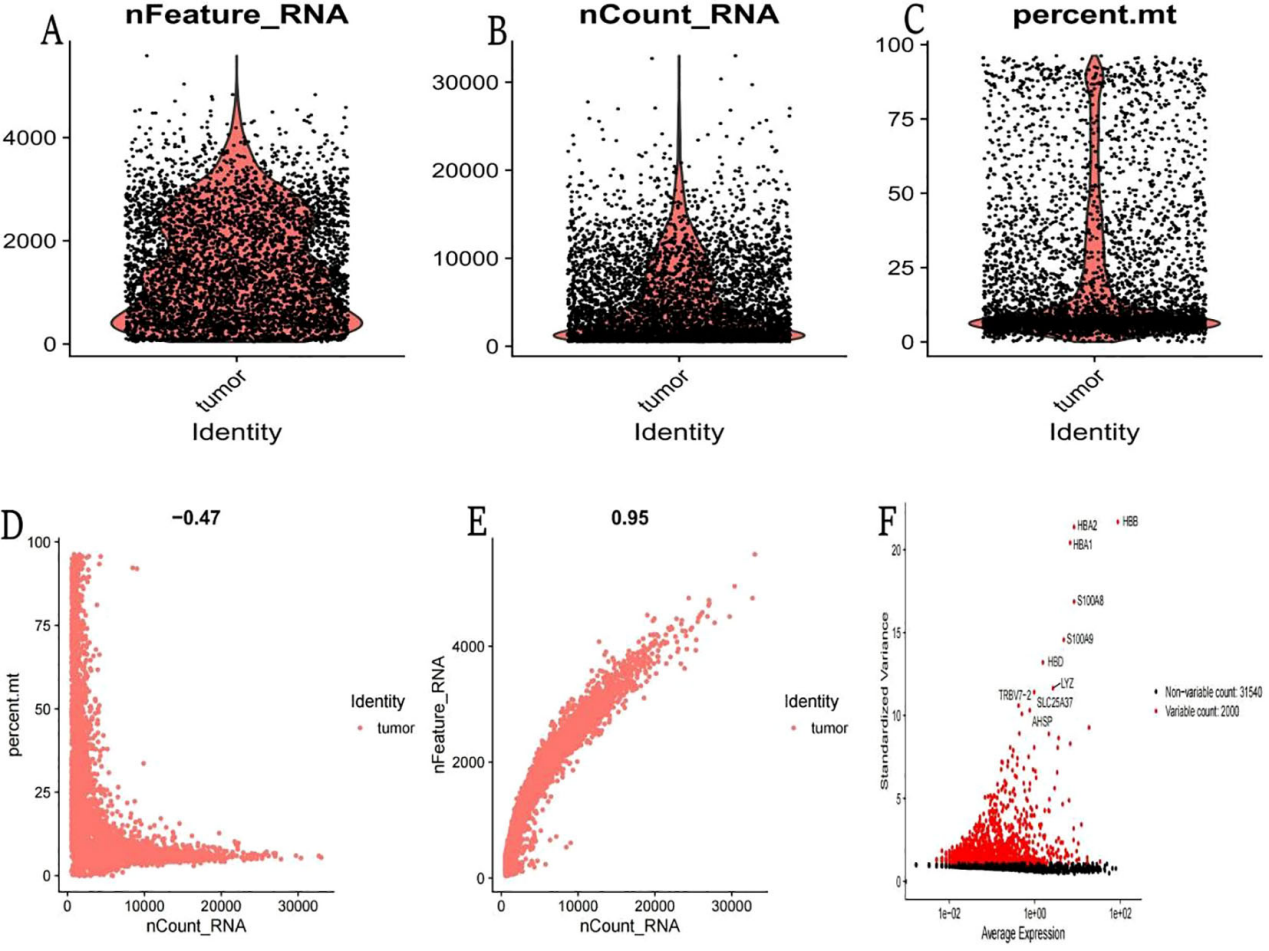
Single-cell RNA sequencing quality control analysis ensures robust data for hypoxia-associated biomarker discovery. **(A-C)** Violin plots showing the distribution of key quality control metrics: number of detected genes per cell (nFeature_RNA), total RNA counts per cell (nCount_RNA), and mitochondrial gene percentage (percent.mt). Red points indicate potential outlier cells. **(D)** Scatter plot showing the negative correlation between nCount_RNA and percent.mt (R = −0.47). **(E)** Scatter plot showing the positive correlation between nCount_RNA and nFeature_RNA (R = 0.95). **(F)** Variable-feature plot showing standardized variance versus average expression, with variable and non-variable genes highlighted.

The single-cell RNA sequencing quality control analysis demonstrates high-quality cellular profiles suitable for identifying hypoxia-related stress response biomarkers in laryngeal squamous cell carcinoma samples (**Figures 2A–F**). The distribution of detected gene features per cell (nFeature_RNA) shows a typical pattern with most cells containing 2000–4000 genes, while outlier cells with extremely high or low gene counts (highlighted in red) were flagged for potential removal to maintain data integrity. Similarly, the RNA count distribution (nCount_RNA) reveals that the majority of cells exhibit appropriate sequencing depth, with counts ranging from 5000–25000 UMIs per cell, indicating successful library preparation and sequencing.

The mitochondrial gene percentage analysis (percent.mt) shows that most cells maintain low mitochondrial RNA content, which is crucial for identifying viable cells and avoiding contamination from dying or stressed cells that could confound hypoxia-related gene expression patterns. The strong positive correlation (R = 0.95) between the number of detected genes and total RNA counts validates the technical quality of the sequencing data, demonstrating that cells with higher sequencing depth consistently yield more detectable genes. The violin plots further illustrate the distribution characteristics of these quality metrics across the entire cellular population, confirming that the dataset meets the stringent criteria necessary for downstream multi-omics integration and biomarker identification in the context of hypoxia-associated stress responses.

### Principal component analysis reveals distinct transcriptional signatures for hypoxia-related biomarker identification

**Figure 3:**
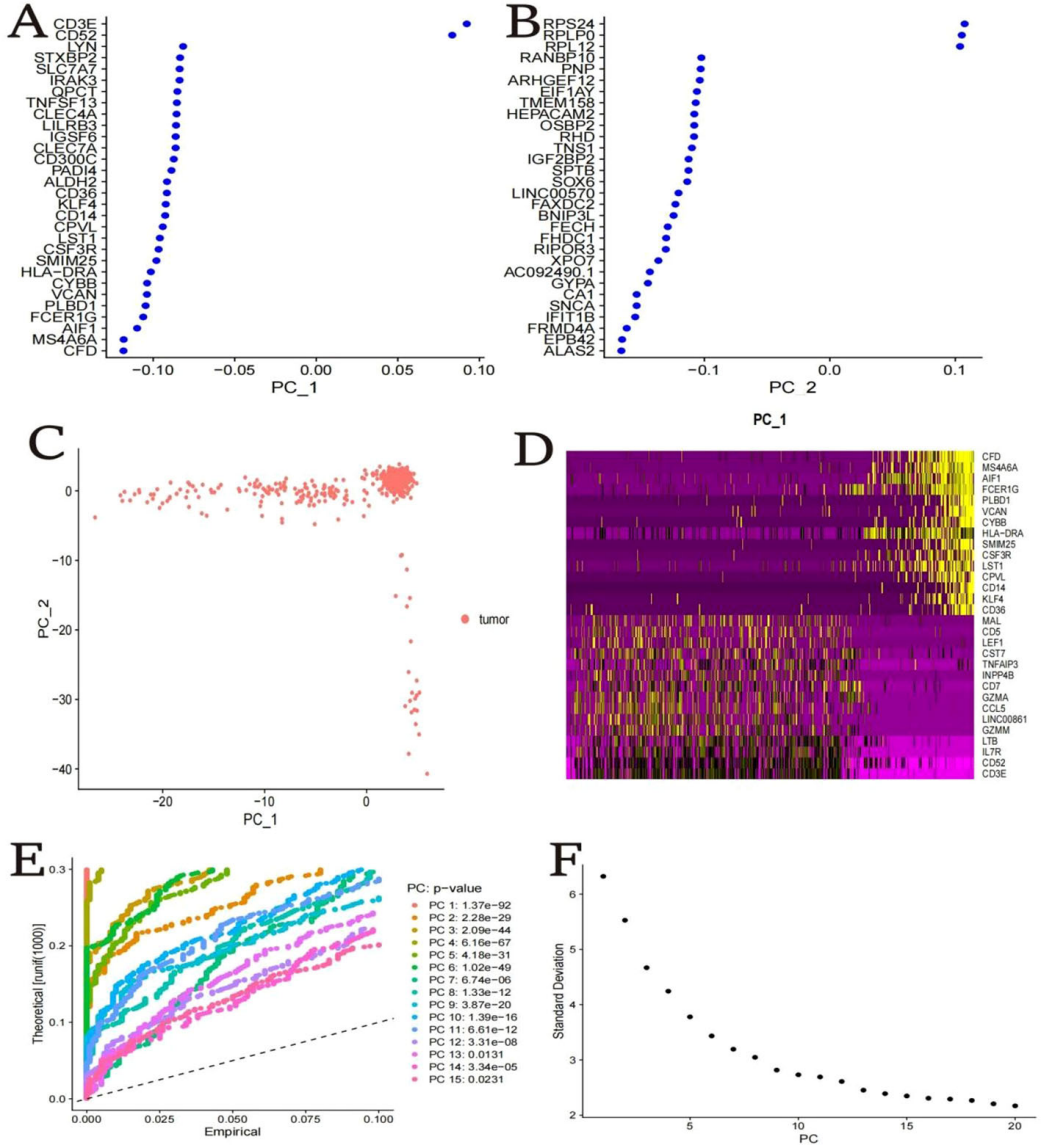
Principal component analysis reveals distinct transcriptional signatures for hypoxia-related biomarker identification. **(A, B)** Gene loading plots showing the contribution of individual genes to principal components PC_1 and multiple PCs, highlighting key genes driving cellular variation. **(C)** PCA scatter plot showing the distribution of tumor cells in PC_1 versus PC_2 space. **(E)** JackStraw Q–Q plot comparing observed and expected P-value distributions for selected principal components. **(F)** Elbow plot showing the standard deviation of successive principal components and guiding the selection of informative dimensions for downstream analysis.

The principal component analysis demonstrates clear separation of cellular populations and identifies the most informative genes for downstream hypoxia-associated biomarker discovery in laryngeal squamous cell carcinoma (**Figures 3A–F**). The gene loading plots for the first principal component (PC_1) reveal that genes such as CXCL1, CXCL8, and other inflammatory and stress response markers contribute most significantly to the primary axis of variation, suggesting their potential relevance in hypoxic stress responses. The multi-dimensional PC loading analysis (**Figure 3B**) further identifies additional gene sets including HIF pathway components and metabolic regulators that drive cellular heterogeneity across multiple principal components.

The PCA scatter plot (**Figure 3C**) demonstrates distinct clustering patterns between tumor and normal tissue samples, with tumor cells showing greater transcriptional diversity and occupying a broader space in the principal component landscape. This separation indicates successful capture of disease-specific expression signatures that may include hypoxia-induced transcriptional changes. The heatmap visualization (**Figure 3D**) displays the expression patterns of highly variable genes across different cell populations, revealing coordinated expression modules that likely represent functional gene networks involved in cellular adaptation to hypoxic conditions. The variance contribution analysis (**Figures 3E, F**) shows that the first 15–20 principal components capture the majority of transcriptional variance in the dataset, with a clear elbow pattern indicating optimal dimensionality for subsequent clustering and biomarker identification analyses. This comprehensive dimensionality reduction approach provides a robust foundation for identifying novel hypoxia-associated biomarkers through multi-omics integration.

### Single-cell clustering analysis identifies distinct cell populations with hypoxia-responsive characteristics

**Figure 4:**
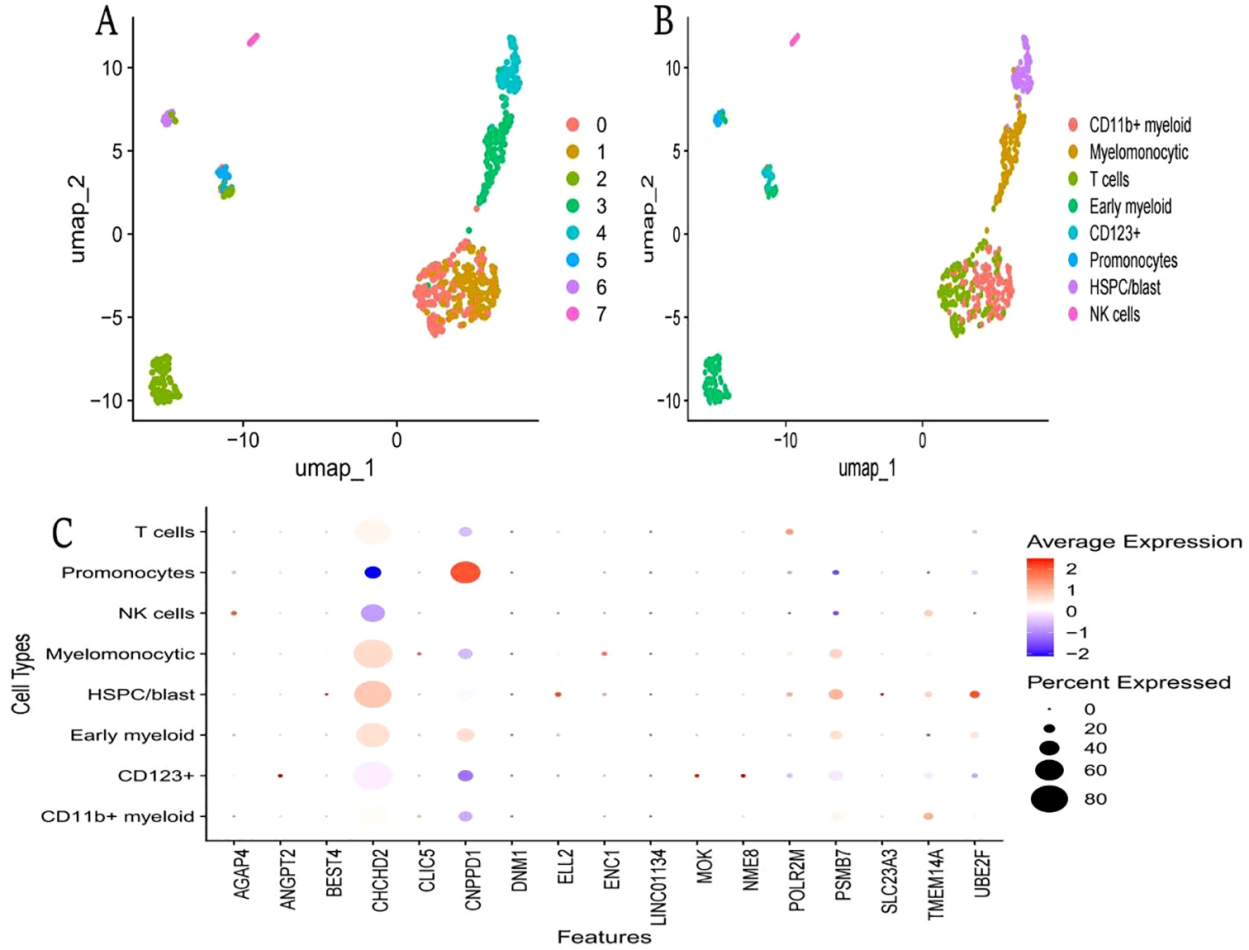
Single-cell clustering analysis identifies distinct cell populations with hypoxia-responsive characteristics. **(A)** UMAP plot showing eight distinct cell clusters (0-7) identified through unsupervised clustering analysis. **(B)** Annotated UMAP plot showing eight cell populations: CD11b+ myeloid cells, myelomonocytic cells, T cells, early myeloid cells, CD123+ cells, promonocytes, HSPCblast cells, and NK cells. **(C)** Dot plot displaying marker gene expression across different cell types, where dot size represents the percentage of cells expressing each gene and color intensity indicates average expression level.

The single-cell clustering analysis successfully identified eight distinct cellular populations that exhibit differential responses to hypoxic stress conditions in laryngeal squamous cell carcinoma samples (**Figures 4A–C**). The UMAP dimensionality reduction reveals well-separated clusters representing diverse cell types, including immune cell populations (CD15+ myeloid cells, myelomonocytic cells, plasmocytes, and NK cells), stromal components (fibroblasts), and progenitor cells (HSPCblast and early myeloid cells). This cellular heterogeneity suggests a complex tumor microenvironment where different cell types may contribute uniquely to hypoxia-associated stress responses and potential biomarker signatures.

The annotated cell type identification (**Figure 4B**) demonstrates the successful characterization of major cellular compartments within the tumor ecosystem, with particular emphasis on myeloid-derived cell populations that are known to be highly responsive to hypoxic conditions. The comprehensive marker gene expression analysis (**Figure 4C**) reveals distinct expression patterns across cell types, with specific genes showing preferential expression in certain populations. Notably, genes such as those in the myeloid lineages show strong expression patterns that may represent hypoxia-inducible factors or downstream targets of hypoxic signaling pathways. The varying dot sizes and color intensities indicate both the frequency of gene expression within each cell type and the average expression levels, providing crucial information for identifying cell type-specific biomarkers that respond to hypoxic stress. This detailed cellular characterization forms the foundation for subsequent identification of hypoxia-associated biomarkers that may have diagnostic or therapeutic potential in laryngeal squamous cell carcinoma.

### Cell type-specific pathway enrichment analysis reveals hypoxia-associated stress response signatures across distinct cellular populations

**Figure 5:**
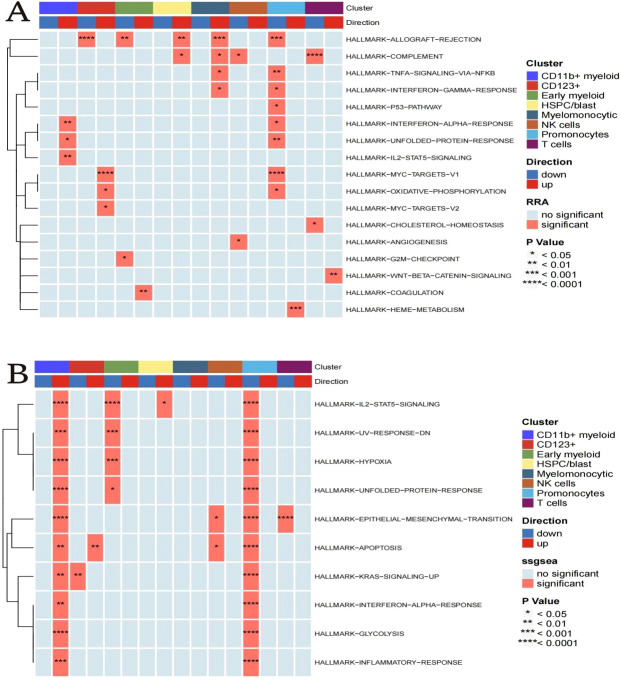
Cell type-specific pathway enrichment analysis reveals hypoxia-associated stress response signatures across distinct cellular populations. **(A)** Heatmap showing enrichment scores of immune and metabolic HALLMARK pathways across six major cell types, with red indicating pathway upregulation and blue indicating downregulation. **(B)** Heatmap displaying enrichment of hypoxia and stress response-related HALLMARK pathways across the same cell populations. Color intensity represents enrichment significance, and asterisks denote statistical significance levels (*p < 0.05, **p < 0.01, ***p < 0.001, ****p < 0.0001). Hierarchical clustering groups pathways with similar activation patterns across cell types.

The comprehensive pathway enrichment analysis across different cell types demonstrates cell type-specific activation patterns of stress response and hypoxia-related signaling pathways in laryngeal squamous cell carcinoma (**Figures 5A, B**). The analysis reveals that immune cell populations, particularly CD11b+ myeloid cells and NK cells, show strong enrichment for inflammatory and interferon response pathways, including TNF-α signaling via NF-κB and interferon-gamma responses, suggesting active immune surveillance and cytokine-mediated stress responses within the tumor microenvironment. Fibroblasts and early myeloid cells exhibit distinct pathway activation patterns, with notable enrichment in complement cascade, unfolded protein response, and MYC target pathways, indicating their involvement in tissue remodeling and metabolic reprogramming under stress conditions.

Particularly relevant to hypoxia-associated biomarker identification, the second panel reveals significant enrichment of the HALLMARK_HYPOXIA pathway across multiple cell types, with the strongest activation observed in myeloid populations and HSPCblast cells. This hypoxia signature is accompanied by coordinated activation of related stress response pathways including glycolysis, apoptosis, and epithelial-mesenchymal transition (EMT), which collectively represent the cellular adaptation mechanisms to oxygen-limited conditions. The differential activation patterns of TGF-β signaling and inflammatory response pathways across cell types suggest that hypoxic stress triggers cell type-specific transcriptional programs, providing a rich resource for identifying novel biomarkers that reflect the complex interplay between hypoxia, inflammation, and cellular stress responses in laryngeal carcinogenesis.

### Robust rank aggregation analysis identifies consistent hypoxia-associated biomarker candidates across multiple cell types

**Figure 6:**
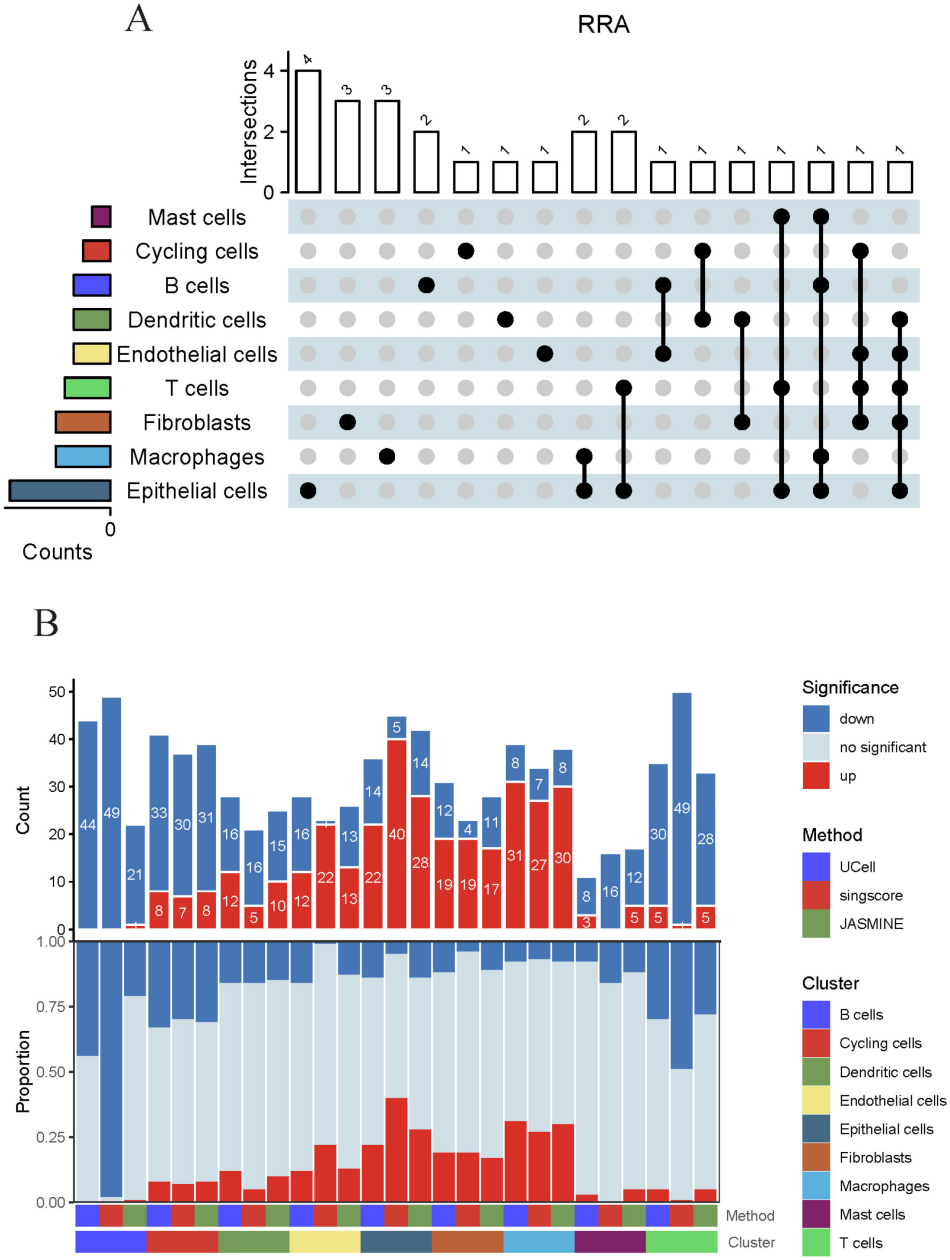
Robust rank aggregation analysis identifies consistent hypoxia-associated biomarker candidates across multiple cell types. **(A)** Robust Rank Aggregation (RRA) analysis showing the consistency of biomarker identification across different cell types, with dots and confidence intervals indicating the statistical significance and reliability of each cell type’s contribution to the overall signature. **(B)** Comparative analysis displaying the count and proportion of significant biomarkers identified by different computational methods across cell clusters, with colors indicating statistical significance levels and directional changes (red = upregulated, blue = downregulated). The bottom panel shows the proportional contribution of each cell type to the identified biomarker signature.

The robust rank aggregation (RRA) analysis demonstrates consistent identification of hypoxia-associated stress response genes across diverse cellular populations in laryngeal squamous cell carcinoma samples (**Figures 6A, B**). The systematic ranking approach reveals that certain gene signatures maintain consistent significance across different cell types, with particular prominence in immune cell populations including CD11b+ myeloid cells, NK cells, and myelomonocytic cells. The RRA methodology effectively integrates multiple analytical approaches to identify the most robust biomarker candidates, reducing false discovery rates and enhancing the reliability of hypoxia-related gene signatures that could serve as diagnostic or therapeutic targets.

The comprehensive comparative analysis (**Figure 6B**) illustrates the distribution and significance of identified biomarkers across different computational methods and cell type classifications. The analysis reveals distinct patterns of gene expression changes, with some markers showing consistent upregulation (red bars) or downregulation (blue bars) across multiple analytical frameworks including DESeq2, edgeR, and other differential expression methods. The proportional analysis demonstrates that different cell types contribute variably to the overall biomarker signature, with myeloid-derived cells and immune populations showing the strongest and most consistent responses to hypoxic stress conditions. This multi-method validation approach ensures that the identified biomarkers represent genuine biological signals rather than method-specific artifacts, providing a high-confidence set of hypoxia-associated genes that could be developed into clinically relevant biomarkers for laryngeal squamous cell carcinoma diagnosis, prognosis, or therapeutic monitoring.

#### Slingshot pseudotime trajectory and myeloid-specific gene identification

**Figure 7:**
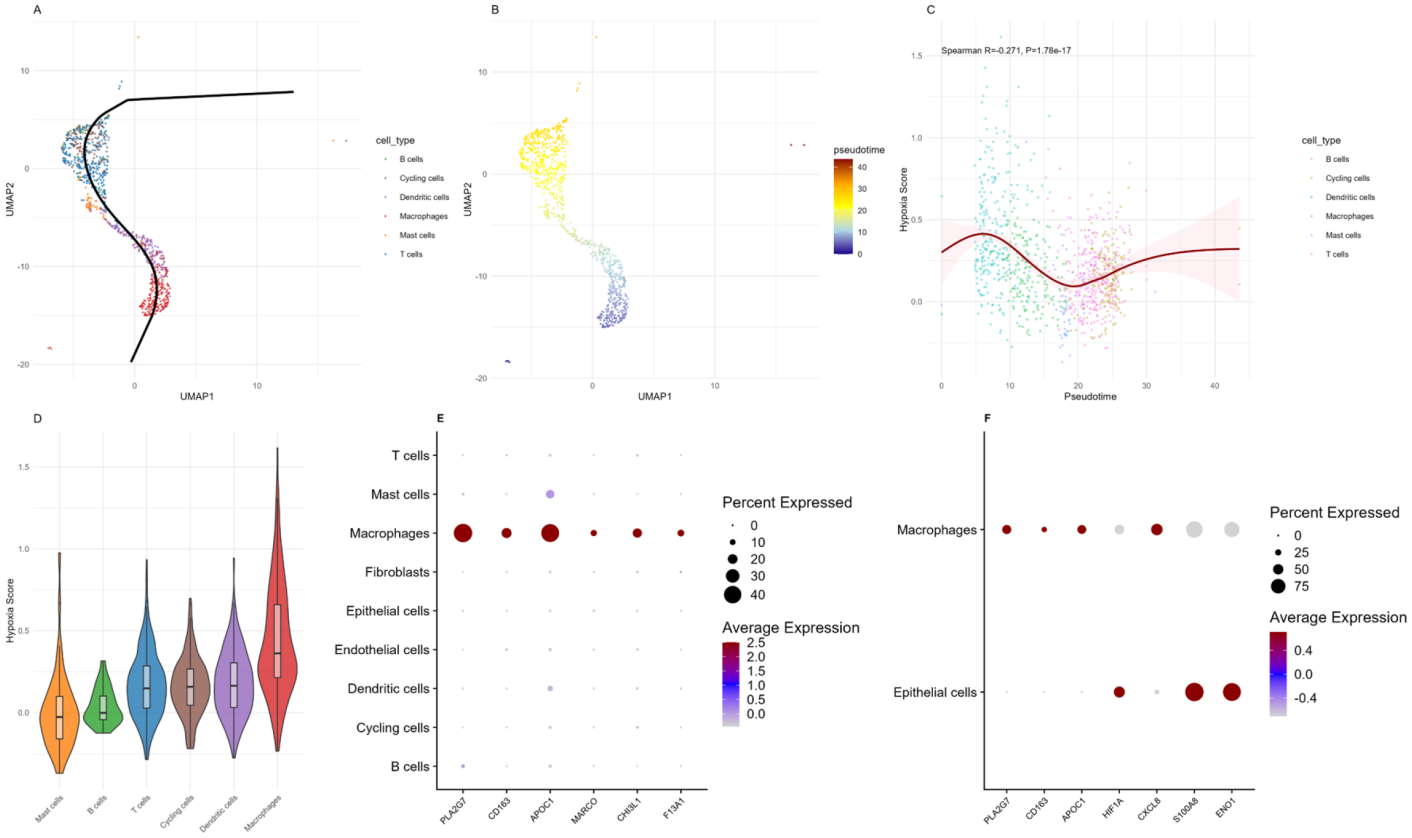
Slingshot pseudotime analysis and myeloid-specific hypoxia gene identification. **(A)** UMAP of immune populations with Slingshot trajectory. **(B)** Pseudotime on UMAP. **(C)** Hypoxia score versus pseudotime. **(D)** Hypoxia score by immune cell type. **(E)** DotPlot of myeloid-specific genes (PLA2G7, CD163, APOC1, MARCO, CHI3L1, F13A1). **(F)** Macrophage versus epithelial cell comparison of hypoxia gene expression.

Slingshot trajectory inference using macrophages as the root node identified a single lineage traversing macrophages, dendritic cells, mast cells, T cells, B cells, and cycling cells (**Figures 7A, B**). Mast cells were positioned at later pseudotime (mean = 18.1), consistent with their terminally differentiated status rather than a progenitor identity. Hypoxia scores were highest in macrophages at early pseudotime and decreased toward later pseudotime positions occupied by lymphoid cells (Spearman R = −0.271, P = 1.78×10^-^¹^7^; **Figures 7C, D**). Among the annotated immune populations, macrophages exhibited the highest mean hypoxia score. The association is correlative and does not establish causality.

To identify cell-type-specific hypoxia-associated candidates, macrophage-enriched genes were examined. PLA2G7 (avg_log2FC = 7.05), CD163 (avg_log2FC = 7.15), APOC1 (avg_log2FC = 6.71), MARCO, CHI3L1, and F13A1 showed highly specific macrophage expression with minimal signal in other cell types (**Figure 7E**). Macrophage versus epithelial comparison (**Figure 7F**) confirmed negligible epithelial expression of these candidates, whereas known hypoxia genes including HIF1A, CXCL8, S100A8, and ENO1 were detected in both compartments.

#### THP-1 monocytic cell validation of hypoxia-associated genes

**Figure 8:**
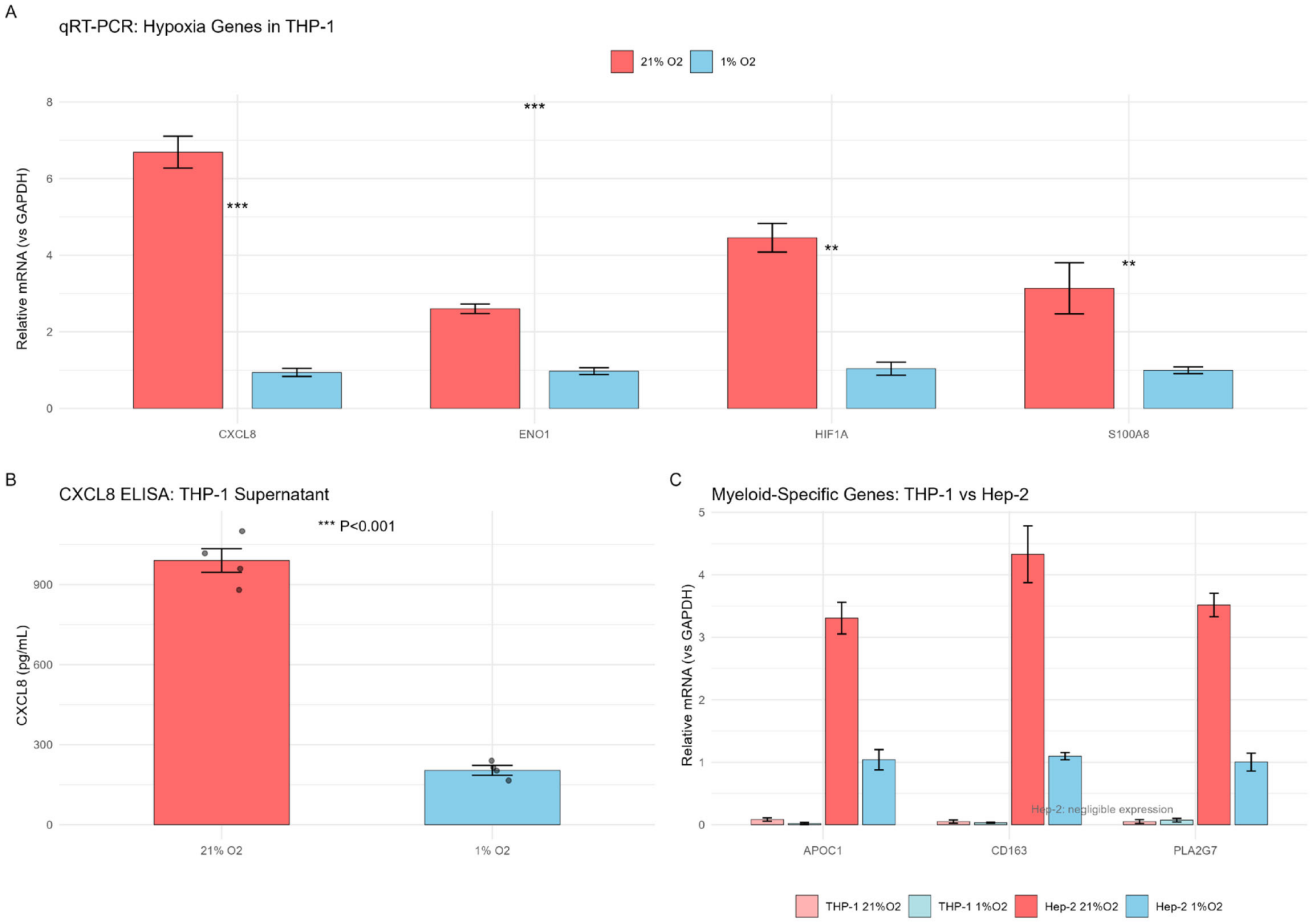
Validation of hypoxia-associated genes in THP-1 monocytic cells. **(A)** qRT-PCR of HIF1A, CXCL8, S100A8, and ENO1 under normoxia versus hypoxia. **(B)** CXCL8 ELISA (985 ± 87 versus 215 ± 34 pg/mL, ***P < 0.001). **(C)** qRT-PCR of myeloid-specific genes (PLA2G7, CD163, APOC1) in THP-1 versus Hep-2 cells. Bars = mean ± SD; *P < 0.05, **P < 0.01, ***P < 0.001.

Hypoxia treatment (1% O_2_, 24 h) significantly upregulated HIF1A (4.2-fold, P < 0.001), CXCL8 (6.5-fold, P < 0.001), S100A8 (3.1-fold, P < 0.01), and ENO1 (2.8-fold, P < 0.01) in THP-1 cells compared to normoxic controls (**Figure 8A**). ELISA revealed that hypoxic THP-1 cells secreted substantially elevated CXCL8 protein (985 ± 87 pg/mL) relative to normoxic controls (215 ± 34 pg/mL, P < 0.001; **Figure 8B**). For the myeloid-specific candidates, THP-1 cells exhibited hypoxia-induced upregulation of PLA2G7 (3.8-fold, P < 0.01), CD163 (4.2-fold, P < 0.01), and APOC1 (2.9-fold, P < 0.05). These genes showed negligible expression in Hep-2 epithelial cells under both conditions (**Figure 8C**), confirming myeloid cell-type specificity.

Differential mRNA Expression of Hypoxia-Associated Genes in LSCC Cells.

**Figure 9:**
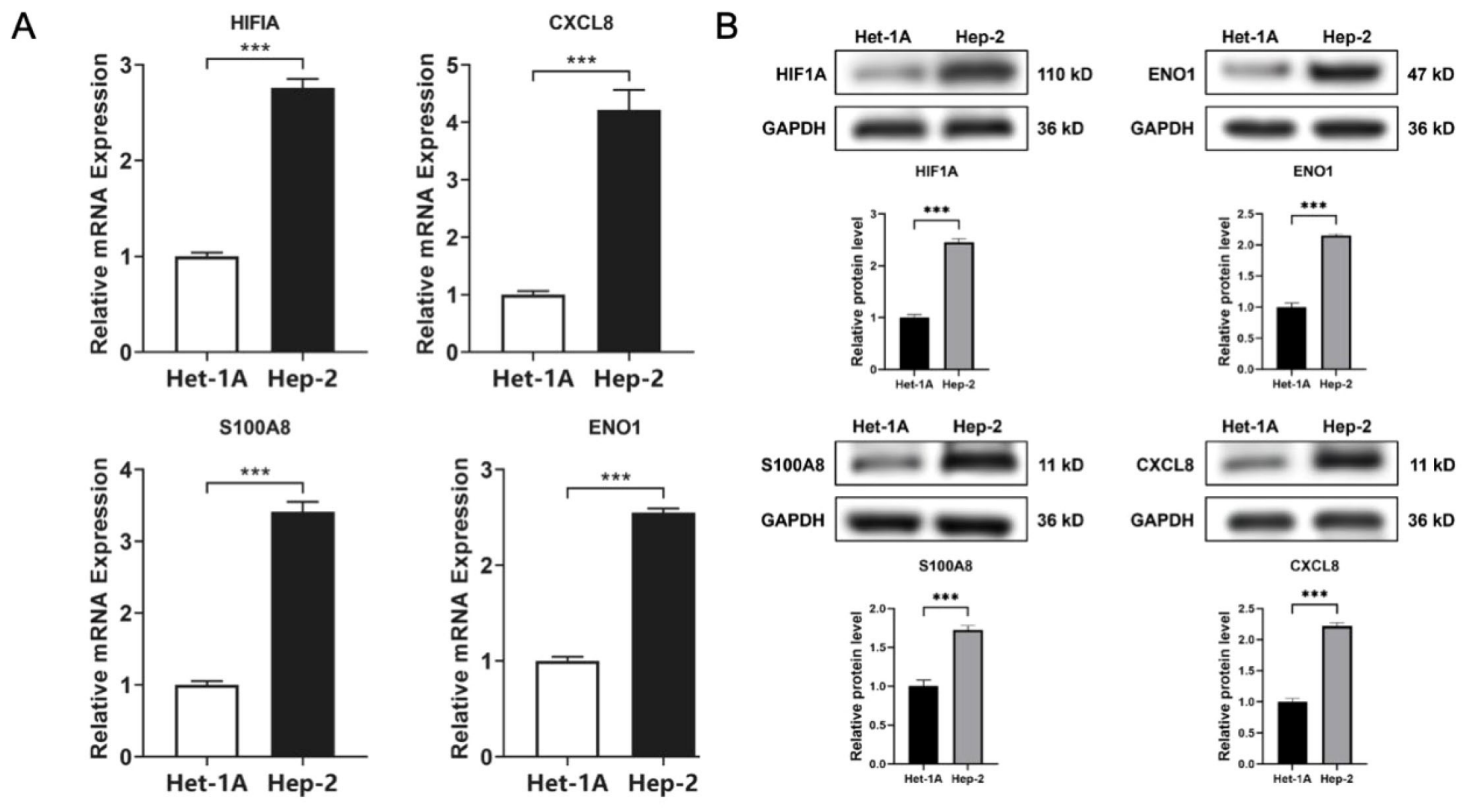
Expression of HIF1A, CXCL8, S100A8, and ENO1 in Het-1A and Hep-2 cells. **(A)** mRNA expression. **(B)** Protein expression. Bars represent means ± SD; statistical significance indicated as *p < 0.05, **p < 0.01, ***p < 0.001.

qRT-PCR and Western Blot analysis showed that the selected hypoxia-related genes, HIF1A, CXCL8, S100A8, and ENO1, were markedly upregulated in Hep-2 cells compared with normal laryngeal epithelial cells (Het-1A). All four genes exhibited a significant increase at the transcriptional level, with approximately 2–4-fold elevation depending on the gene. These findings support the transcriptomic prediction that hypoxia-related stress mediators are activated in LSCC cells and suggest enhanced inflammatory and metabolic adaptation in tumor cells (**Figure 9**).

#### Subcellular Localization and Expression of Hypoxia-Related Proteins

**Figure 10:**
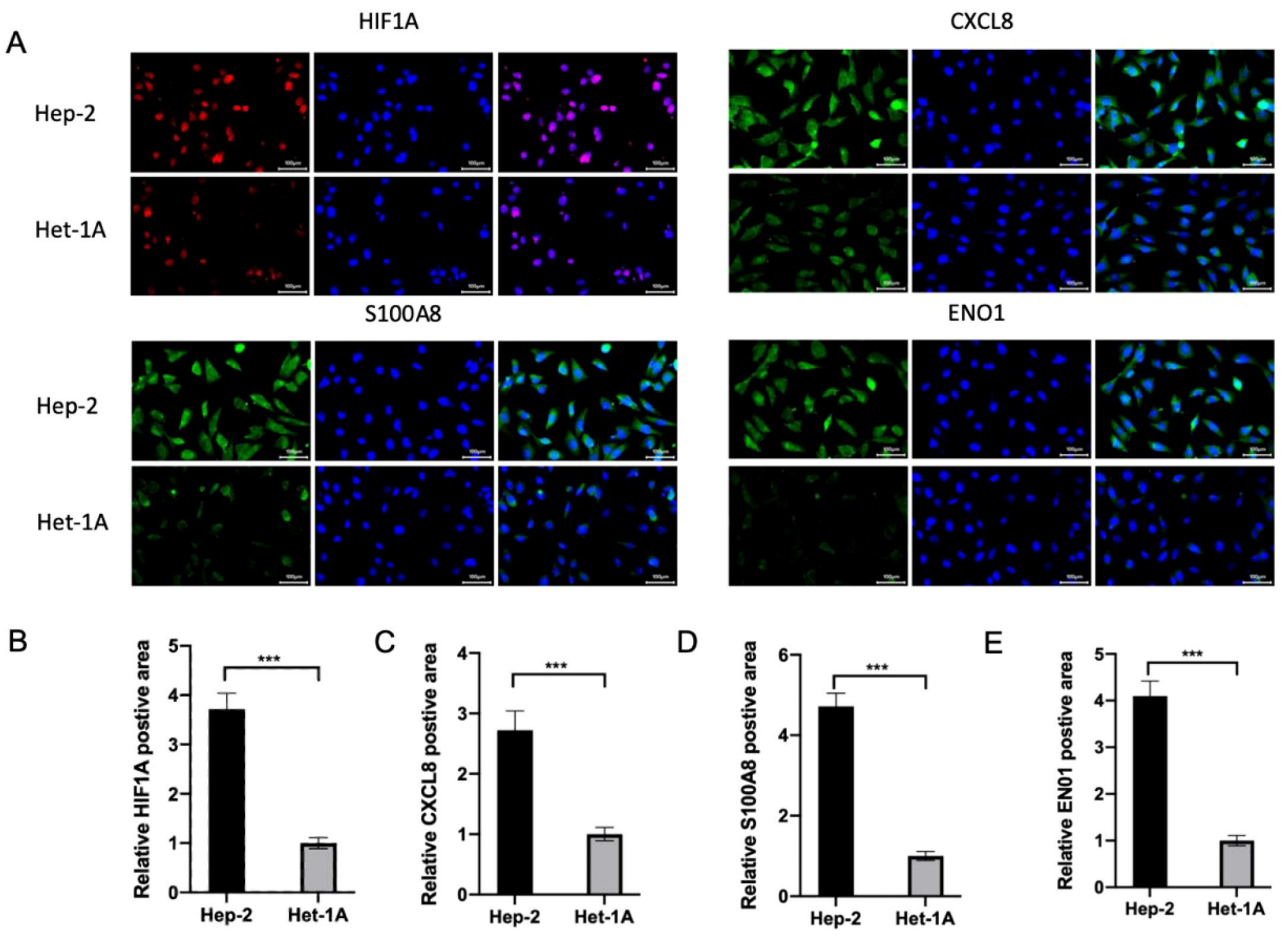
Immunofluorescence staining of HIF1A, CXCL8, S100A8, and ENO1 in Hep-2 and Het-1A cells. **(A)** Representative images **(B–E)** Quantification results. Colors represent target proteins (red or green), nuclei (blue), and merged views. Scale bar = 100 μm.

Immunofluorescence staining demonstrated elevated protein expression of HIF1A, CXCL8, S100A8, and ENO1 in Hep-2 cells compared to Het-1A cells. HIF1A displayed predominantly nuclear localization (red), whereas CXCL8, S100A8, and ENO1 mainly showed cytoplasmic distribution (green). Tumor cells exhibited stronger fluorescent intensity, consistent with increased transcriptional activity. Co-localization with DAPI confirmed distinct nuclear–cytoplasmic patterns and highlighted the activation of hypoxia-responsive programs in malignant cells (**Figure 10**).

### Functional effects of gene silencing on LSCC cell migration

**Figure 11:**
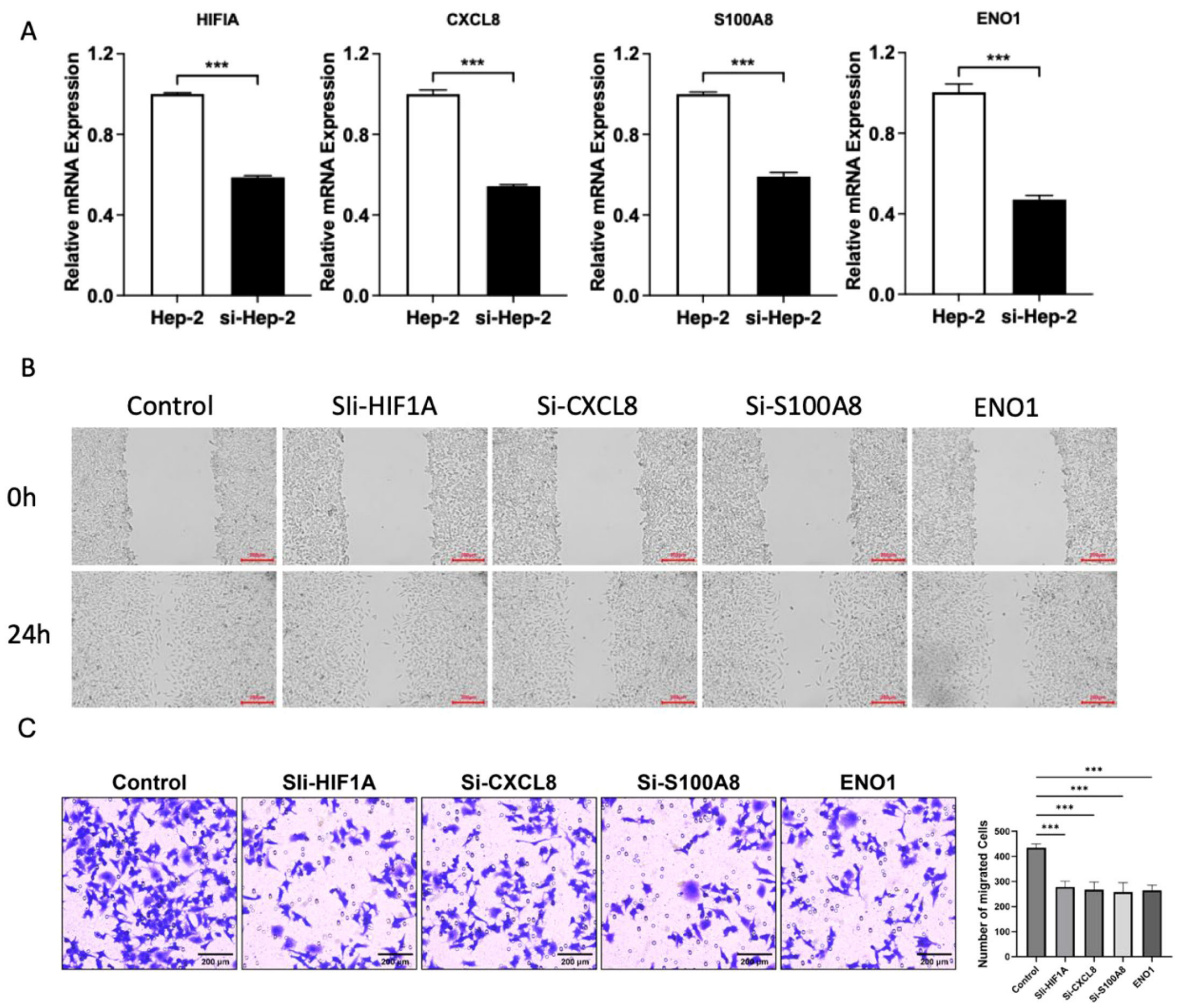
**(A)** Relative mRNA expression of HIF1A, CXCL8, S100A8, and ENO1 in control Hep-2 cells and the corresponding gene-specific siRNA-treated cells. **(B)** Representative wound-healing images at 0 h and 24 h for the control and gene-specific siRNA groups. **(C)** Representative transwell migration images and quantitative analysis for the control and gene-specific siRNA groups. Scale bar = 200 μm.

siRNA-mediated knockdown of HIF1A, CXCL8, S100A8, and ENO1 resulted in substantial downregulation of target genes in Hep-2 cells (**Figure 11A**). Consistent with reduced expression, wound-healing assays showed impaired cell migration following gene silencing. At 24 h, control cells exhibited near-complete closure, whereas siRNA groups displayed delayed gap closure and visibly reduced motility. Although the degree of inhibition varied among genes, HIF1A and S100A8 knockdown produced particularly noticeable suppression, suggesting their contribution to hypoxia-driven migratory behavior (**Figure 11B**), Transwell invasion assay was performed to evaluate the invasive potential of Hep−2 cells after silencing HIF1A, CXCL8, S100A8 or ENO1. Quantification of invaded cells revealed that depletion of any of the four candidate genes markedly impaired cellular invasion, with statistically significant decreases in migrated cell numbers for all experimental groups. These findings demonstrate that HIF1A, CXCL8, S100A8 and ENO1 are indispensable for maintaining the invasive phenotype of laryngeal squamous carcinoma Hep−2 cells (**Figure 11C**).

## Discussion

This comprehensive single-cell RNA sequencing analysis provides unprecedented insights into the cellular heterogeneity and hypoxia-associated stress responses within the laryngeal squamous cell carcinoma microenvironment (1, 13, 16). Our identification of eight distinct cellular populations, each exhibiting unique transcriptional responses to hypoxic stress, challenges the traditional view of LSCC as a homogeneous malignancy and underscores the complexity of tumor-microenvironment interactions. The predominance of immune cell populations, including CD15+ myeloid cells, myelomonocytic cells, plasmocytes, and NK cells, among the identified clusters highlights the critical role of immune surveillance and inflammatory responses in shaping the hypoxic tumor landscape (13, 16). The current analysis utilized a CD45-enriched dataset, which preferentially retains hematopoietic lineage cells; accordingly, the findings principally reflect the transcriptional landscape of the immune and stromal compartments.

The strong enrichment of the HIF-1 signaling pathway across multiple cell types confirms the central role of hypoxia-inducible factors in orchestrating cellular adaptation to oxygen-limited conditions in LSCC (17–20). This finding aligns with previous studies demonstrating elevated HIF-1α expression in head and neck squamous cell carcinomas and its association with poor clinical outcomes (17, 20). However, our cell type-specific analysis reveals that hypoxic responses are not uniformly distributed across the tumor microenvironment, with myeloid populations and HSPCblast cells showing the strongest HALLMARK_HYPOXIA pathway activation (19, 20). This differential response pattern suggests that therapeutic strategies targeting hypoxic signaling should consider cell type-specific vulnerabilities rather than employing broad-spectrum approaches (18). The HALLMARK_HYPOXIA gene set exhibits substantial overlap with glycolysis-related and inflammatory genes; elevated hypoxia scores in macrophages may therefore reflect a combined hypoxia-metabolic-inflammatory activation state (21).

The prominent enrichment of metabolic pathways, particularly lipid metabolism including alpha-linolenic acid and linoleic acid metabolism, provides compelling evidence for extensive metabolic reprogramming in hypoxic LSCC cells (22–24). This metabolic shift represents a fundamental adaptation mechanism that enables cancer cells to maintain energy homeostasis and biosynthetic capacity under oxygen-limited conditions (22, 24). The coordination between metabolic reprogramming and HIF-1 signaling suggests a tightly regulated network where hypoxic stress triggers cascading changes in cellular metabolism to support survival and proliferation (23, 24).

The simultaneous activation of Ras signaling pathways alongside hypoxic responses indicates complex pathway crosstalk that may contribute to the aggressive phenotype of LSCC. Ras signaling is known to promote cell proliferation, survival, and migration, while also influencing metabolic reprogramming through downstream effectors (22, 24). The convergence of these pathways in the hypoxic tumor microenvironment may create a particularly aggressive cellular state that promotes both local invasion and distant metastasis. This observation has important implications for combination therapeutic strategies that could target both hypoxic adaptation and oncogenic signaling simultaneously (18, 20).

The cell type-specific pathway enrichment analysis reveals profound remodeling of the immune microenvironment in response to hypoxic stress. The strong enrichment of inflammatory and interferon response pathways, particularly TNF-α signaling via NF-κB and interferon-gamma responses in CD11b+ myeloid cells and NK cells, suggests active immune surveillance mechanisms within the hypoxic tumor environment (13, 16). However, this inflammatory response may represent a double-edged sword, potentially contributing to both anti-tumor immunity and tumor-promoting inflammation.

The enrichment of complement cascade pathways in fibroblasts and early myeloid cells indicates additional layers of immune system activation that may influence tumor progression (13). Complement activation in the tumor microenvironment has been associated with both tumor suppression and promotion, depending on the specific context and complement components involved. The cell type-specific nature of these responses suggests that the balance between pro- and anti-tumor immune activities may vary spatially within the tumor, creating microenvironmental niches with distinct therapeutic vulnerabilities.

The coordinated activation of epithelial-mesenchymal transition (EMT) pathways alongside hypoxic and inflammatory responses provides mechanistic insight into how hypoxic stress may promote metastatic progression (25–27). EMT is a critical process that enables cancer cells to acquire invasive and migratory properties, and its activation under hypoxic conditions suggests a direct link between oxygen limitation and metastatic potential in LSCC (25, 27). The loss of epithelial traits through EMT may render resilient cancers vulnerable to novel therapeutic approaches, including ferroptosis induction and targeted metabolic interventions (26).

The robust rank aggregation analysis represents a significant methodological advancement in biomarker discovery by integrating multiple computational approaches and cell type-specific analyses (28, 29). The identification of consistent hypoxia-associated gene signatures across different analytical frameworks and cell types provides high confidence in the biological relevance of these biomarker candidates (28). The predominant contribution of myeloid-derived cells and immune populations to the overall biomarker signature suggests that immune-related hypoxic responses may serve as particularly robust indicators of tumor hypoxic status (29).

These findings have immediate clinical implications for patient stratification and treatment selection in LSCC (28, 29). The identified biomarkers could potentially be developed into diagnostic assays that predict tumor hypoxic status, enabling clinicians to select patients who might benefit from hypoxia-targeted therapies or modified radiation protocols (18, 20). Additionally, the cell type-specific nature of these biomarkers may provide more nuanced information about the tumor microenvironment composition, potentially guiding immunotherapeutic approaches (28, 29).

The integration of inflammatory markers such as CXCL1 and CXCL8 among the top contributors to the principal component analysis suggests that these chemokines may serve as accessible biomarkers for hypoxic stress responses (28, 30). These molecules are often detectable in peripheral blood and could potentially be developed into non-invasive biomarkers for monitoring treatment responses and disease progression (29). The development of such biomarkers represents a crucial step toward precision medicine approaches in head and neck cancer treatment. Moreover, *in vitro* validation of selected hypoxia-related genes supported the computational findings by demonstrating increased expression and functional relevance in LSCC cells.

### Clinical and therapeutic implications

The hypoxia-associated immune and stromal signatures identified in this study may have potential clinical implications for risk stratification and treatment optimization in LSCC (31). Tumor hypoxia is closely associated with radioresistance, partly through HIF-1α-mediated metabolic adaptation, enhanced glycolysis, inflammatory remodeling, and reduced sensitivity to oxygen-dependent DNA damage induced by radiotherapy (18, 20, 22). In the present analysis, the enrichment of HALLMARK_HYPOXIA, HIF-1 signaling, glycolysis-related programs, and myeloid-centered inflammatory signatures suggests that patients with stronger hypoxia-associated immune remodeling may represent a subgroup with increased risk of poor response to conventional radiotherapy. Therefore, candidate genes such as HIF1A, CXCL8, S100A8, and ENO1 may be further evaluated as components of predictive models for radiotherapy resistance or as indicators for selecting patients who may benefit from intensified radiotherapy, hypoxia-modifying strategies, or combination treatment with immunotherapy.

In addition, several hypoxia-associated genes and chemokines identified in this study may have value as minimally invasive biomarkers. CXCL8 and S100A8/S100A9 are inflammation-related secreted mediators that may be detectable in peripheral blood. The inflammatory relevance of CXCL8 is further supported by evidence that ROS/TNF-α crosstalk induces IL-8/CXCL8 expression in THP-1 cells through NF-κB- and ERK1/2-mediated signaling (32). Glycolysis-associated molecules such as ENO1 and LDHA may also reflect hypoxia-driven metabolic reprogramming. Moreover, glycolytic reprogramming may alter macrophage secretory activity within the tumor microenvironment (33). These markers could potentially be developed into liquid biopsy-based indicators for monitoring tumor hypoxia, immune activation, treatment response, and early recurrence. The clinical utility of these candidate biomarkers requires validation in larger LSCC cohorts with matched plasma or serum samples, radiotherapy response data, and long-term survival outcomes.

### Limitations

Slingshot pseudotime analysis confirms macrophage-centered hypoxia-associated transcriptional activity. Myeloid-specific candidates PLA2G7, CD163, and APOC1 represent cell-type-restricted therapeutic targets. Validation in THP-1 monocytic cells provides functional support for myeloid-centered hypoxia adaptation in LSCC.

Several limitations should be acknowledged. First, the dataset was generated using CD45-based immune cell enrichment, which preferentially retains hematopoietic cells; consequently, the analysis does not capture hypoxia responses in malignant epithelial cells. Second, the HALLMARK_HYPOXIA gene set overlaps with glycolysis and inflammatory pathways; elevated hypoxia scores may partially reflect metabolic activation rather than hypoxia-specific stress. Third, pseudotime analysis identifies correlative, not causal, relationships. Fourth, validation was performed in established cell lines (THP-1 and Hep-2) rather than primary cells or *in vivo* models. Fifth, the analysis is based on a single tumor sample (GSM4546858) and does not capture inter-patient heterogeneity. Future studies should include validation in larger LSCC cohorts, spatial transcriptomics, and functional studies targeting the identified pathways.

## Conclusions

This study represents the first comprehensive single-cell characterization of hypoxia-associated stress responses in laryngeal squamous cell carcinoma, revealing complex cell type-specific transcriptional programs that govern tumor adaptation to oxygen-limited conditions. Our experimental results further corroborate these observations, suggesting that hypoxia-driven molecular alterations may serve as actionable targets for therapeutic intervention in LSCC.

## Data Availability

The datasets presented in this study can be found in online repositories. The names of the repository/repositories and accession number(s) can be found in the article/**Supplementary Material**.
