# Supplementary material for: Single-cell RNA sequencing reveals hypoxia-associated stress response signatures in the immune and stromal microenvironment of laryngeal squamous cell carcinoma
Source: Front Immunol. 2026 Aug 4;17:1850654. doi: 10.3389/fimmu.2026.1850654 (PMC13481874; doi:10.3389/fimmu.2026.1850654)
Supplement: Supplementary file 1 [file Table1.docx]

Supplementary Table S1: RRA Screening Candidate Gene Table for Hypoxia-Related Genes

| **Gene symbol** | **Combined RRA P-value** | **RRA rank** | **Up/Down regulated** |
| --- | --- | --- | --- |
| HIF1A | 2.12E-06 | 1 | Up |
| CXCL8 | 3.45E-06 | 2 | Up |
| S100A8 | 5.18E-06 | 3 | Up |
| ENO1 | 7.33E-06 | 4 | Up |
| CXCL1 | 1.24E-05 | 5 | Up |
| S100A9 | 1.86E-05 | 6 | Up |
| LDHA | 2.51E-05 | 7 | Up |
| PGK1 | 3.02E-05 | 8 | Up |
| ALDOA | 3.77E-05 | 9 | Up |
| MMP9 | 4.21E-05 | 10 | Up |
